# Microstructure and Mechanical Properties of Underwater Laser Welding of Titanium Alloy

**DOI:** 10.3390/ma12172703

**Published:** 2019-08-23

**Authors:** Ning Guo, Qi Cheng, Xin Zhang, Yunlong Fu, Lu Huang

**Affiliations:** 1 State Key Laboratory of Advanced Welding and Joining, Harbin Institute of Technology, Harbin 150000, China; 2 Shandong Provincial Key Laboratory of Special Welding Technology, Harbin Institute of Technology at Weihai, Weihai 264209, China; 3 Shandong Institute of Shipbuilding Technology, Weihai 264209, China

**Keywords:** underwater laser beam welding, Ti-6Al-4V alloy, local dry cavity, welding surface appearance, microstructure, mechanical properties

## Abstract

Underwater laser beam welding (ULBW) with filler wire was applied to Ti-6Al-4V alloy. Process parameters including the back shielding gas flow rate (BSGFR) (the amount of protective gas flowing over the back of the workpiece per unit time), focal position, and laser power were investigated to obtain a high-quality butt joint. The results showed that the increase of BSGFR could obtain the slighter oxidation level and refiner crystal grain in the welded metals. Whereas the back shielding gas at a flow rate of 35 L/min resulting in pores in the welded metals. With the increasing of the heat input, the welded metals went through three stages, i.e., not full penetration, crystal grain refinement, and coarseness. Crystal grain refinement could improve the mechanical properties, however, not full penetration and pores led to the decline in mechanical properties. Under optimal process parameters, the microstructure in the fusion zones of the underwater and in-air weld metals was acicular martensite. The near the fusion zone of the underwater and in-air weld metals consisted of the α + α′ phase, but almost without the α′ phase in the near base metal zone. The tensile strength and impact toughness of the underwater welded joints were 852.81 MPa and 39.07 J/cm^2^, respectively, which approached to those of the in-air welded joints (861.32 MPa and 38.99 J/cm^2^).

## 1. Introduction

Titanium and its alloys have many advantages, such as high specific strength, outstanding corrosion resistance, and superior biocompatibility, so they are extensively applied to aerospace field, ocean engineering, and biomedical devices. Kumar et al. researched the tungsten inert gas (TIG) welding of Ti-6Al-4V alloy [1]. Beguin et al. explored the laser welding of thin titanium sheet [2]. Chen et al. carried out laser welding with filler wire of Ti-alloy [3].

Underwater welding (UW) turns into the dominating means of maintenance, which was used in offshore pipelines and platforms, vessels, seashore components, as well as port equipment and systems [4]. As reported by Shi and Łabanowski, there are three kinds of underwater welding processes, including underwater dry welding (UDW), underwater wet welding (UWW) and underwater local dry cavity welding (ULDCW) [4,5]. UDW is usually implemented in an underwater high-pressure chamber and can obtain high-quality welded joints, but in comparison to UWW, the welding equipment of UDW is complex and expensive, extremely [6]. Hence, UWW technology is generally employed [7]. Rogalski et al. pointed out that UWW could maintain cracks and corrosion [8]. Guo et al. explored the forming mechanism of spatter, the stability and the pulsating wire feeding process of underwater wet arc welding (UWAW) with flux-cored wires based on the X-ray transmission method [7,9,10]. Pessoa et al. indicated the porosity formed owing to water vapor, molecular hydrogen, or carbon monoxide during UWW [11]. In comparison to underwater arc welding (UAW), ULBW has some significant advantages, for instance, more convenience, precise heat input, and a completely automatic welding process [12]. Shannon et al. investigated the direct ULBW, the result indicated that the Nd:YAG laser was unfeasible, while the CO_2_ laser was suitable for direct ULBW [13]. Yoda et al. deemed the direct ULBW could get over some defects of UWAW and studied the feasibility of the direct ULBW [14]. Guo et al. reported the stable ‘beam channel’ was the key to conducting the direct ULBW [15]. During ULDCW, the water of a small scope is drained to form a local dry cavity, making the welding process steady. Therefore, in comparison to UWW, this means it can obtain higher-performance welded joints, besides, it is cheaper and more convenient than UDW [4]. Shi et al. investigated the welded joints of high strength steel via UDW and ULDCW. It was found that the performance of ULDCW joints was worse compared with that of UDW joints on account of the high cooling rate [4]. Cui et al. studied the welded joints of duplex stainless steel by ULDCW. The result showed that the impact toughness met the requirements of UW criterions and the ratio of austenite reduced in the underwater weld metals compared with the in-air weld metals [16]. Zhang et al. studied the influence of shielding conditions and optical signals on the quality of weld metals during ULBW, respectively [17,18]. Guo et al. indicated that the high-quality welded joints of ULBW were obtained under appropriate welding parameters by using a double-layer gas curtain nozzle [12]. 

However, owing to the spot size of the laser beam was small, it was difficult to adjust the laser beam parameters and align the samples, besides, under the condition of high-speed laser welding of titanium alloy, there were generally different degrees of edgewise defects in the weld metal, which would seriously affect the mechanical properties of the welded joint. In order to figure out the aforementioned problems, ULBW with filler wire was investigated [19]. At the same time, this process also had some shortcomings, for example, due to the addition of welding wire, the interaction of laser, welding wire, and base metal became more complex; the distance between the laser spot center and welding wire center should be controlled in a very small range. Zhang et al. studied that ULBW with filler wire of 304 stainless steel. The result manifested that the mechanical properties of the underwater and in-air welded joints were approximately the same under suitable shielding conditions [20].

While ULBW with filler wire of Ti-6Al-4V alloy has received little focused attention so far. In this thesis, the welding surface appearance, microstructure, and mechanical properties of different process parameters were researched by the self-designed double-layer gas-protective cover. Moreover, the influence of the water environment on the welding quality at optimum welding parameters was also investigated. The research results provided the technical basis for underwater emergency maintenance of titanium alloy ships.

## 2. Materials and Methods

**Figure 1:**
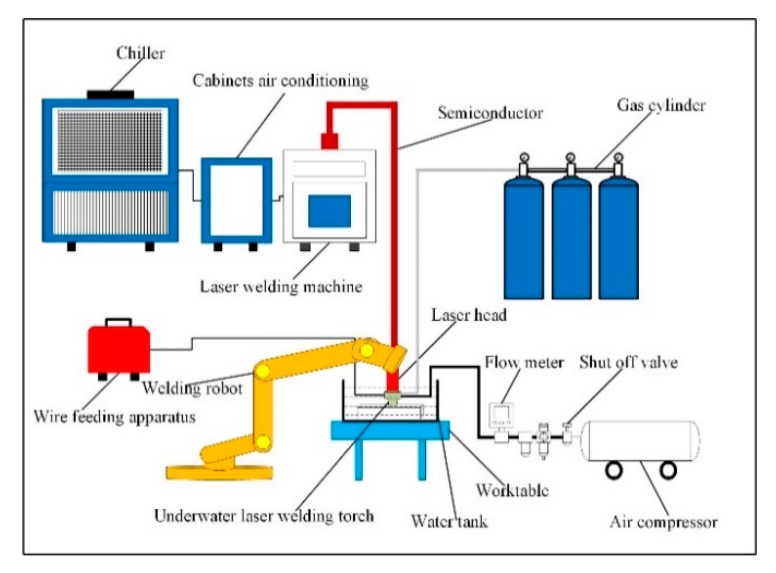
ULBW with filler wire experiment platform.

**Figure 2:**
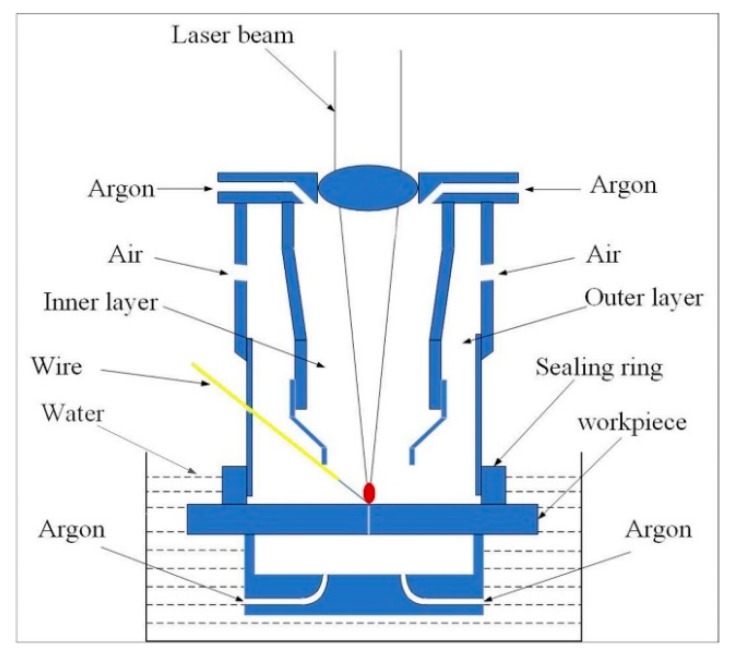
The schematic diagram of the double-layer gas-protective cover.

The experimental platform of ULBW with filler wire is shown in Figure 1. In this experiment, the power of the semiconductor laser (Made by Han’s Laser, at Shenzhen, China) was 5 kW, the wavelength was 915 nm, and the optical fiber core diameter was 800 μm. The welding process was conducted via a six-axial welding robot (Made by Yaskawa Electric Co., Ltd., at Shanghai, China). In addition, the welding wire and shielding gas were placed in front of the laser, and the included angle between the welding wire and the workpiece was 45°. The schematic diagram of the double-layer gas-protective cover is displayed in Figure 2. During the welding process, the whole double-layer gas protective cover was fixed under the laser head by the specific fixture, the outer layer was full of air by using an air compressor in order to exclude the water, at the same time, the inner layer brimmed with the ultrahigh purity argon for the sake of driving the air away and protecting the weld metal, which was essential to providing both quality superiorities and cost benefits. In addition, the back shielding gas was supplied by ultrahigh purity argon to prevent workpiece oxidation at high temperatures.

**Figure 3:**
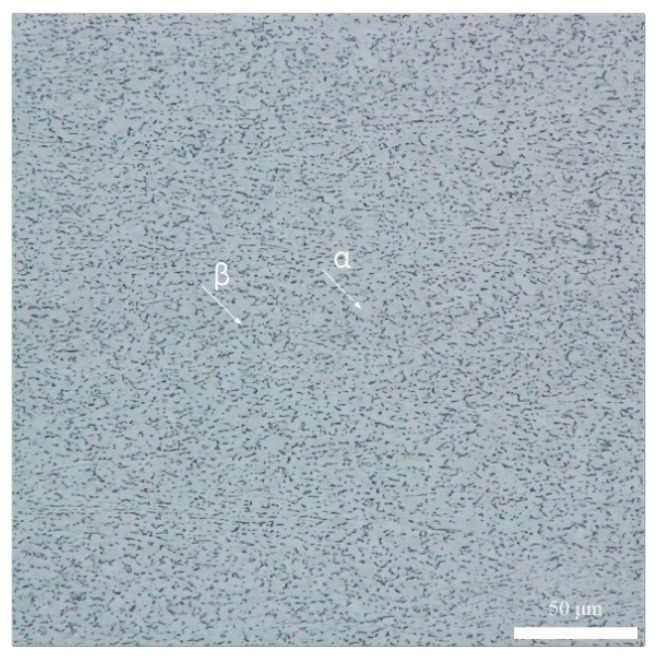
The microstructure of the base metal.

The Ti-6Al-4V alloy thin sheets of 2.0 mm thickness were selected as the base metal in this research, whose chemical component is shown in Table 1, Table 2 listed the mechanical properties of the base metal. In addition, the diameter of the Ti-6Al-4V alloy welding wire was 1.0 mm during welding. The microstructure of the base metal at room temperature is shown in Figure 3, it could be found that the base metal was composed of equiaxial α phase and intergranular distribution of β phase. It found that the α phase was white, the β phase was black. Besides, the β phase evenly distributed around the α phase. 

Throughout the experiments, the welding processes were carried out at the different back shielding gas flow rates, focal positions, and laser powers. Table 3 displayed the concrete welding parameters.

**Figure 4:**
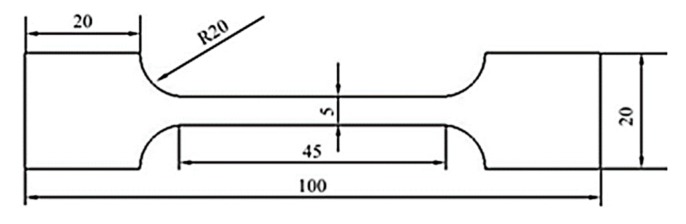
Schematic diagram of the tensile sample size. unit: mm.

**Figure 5:**
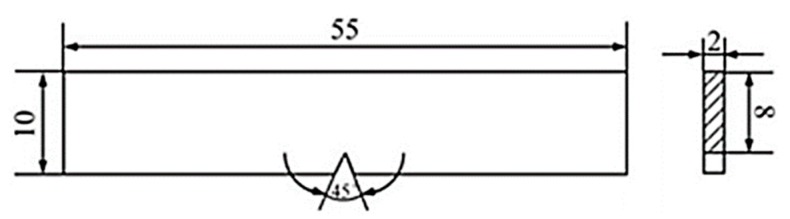
Schematic diagram of the impact sample size. unit: mm.

After welding, the swatch was cut along the direction perpendicular to the weld metal. Afterwards, the swatch was mechanically polished with the metallographic sandpaper and the aluminum oxide polishing agent. After polishing, it was etched via using the Kroll reagent (100 mL H_2_O + 1.5 mL HF + 3 mL HNO_3_) until the surface of the samples were grayish black. The microstructures of the weld metals were observed by the light microscope (Made by Olympus Corporation, at Tokyo, Japan), then the Vickers microhardness indentation machine (Made by Shanghai Gaozhi Precision Instrument Co., Ltd., at Shanghai, China) was used to test the Vickers microhardness, whose test load and dwell time were 200 g and 10 s, respectively, the measuring lines in the middle of the welded joints from the end to another end, the distance of two adjacent test points was 0.25 mm. Three tensile specimens were cut for the each welded joint, with the objective of obtaining tensile properties, the dimensions of the tensile sample were exhibited in Figure 4, the specimens were measured by means of the Instron 5967 type 30 kN universal material testing machine (Made by Instron Corporation, at London, UK) at room temperature, whose tensile speed was 1.0 mm/min and tensile strength depending on the mean value of three samples. Analogously, each of the welded joints sliced three impact samples, the dimensions of the impact sample were exhibited in Figure 5. The swatch was tested via the standard Charpy V-notch impact test at room temperature. In the end, the fractures were surveyed to obtain the surfaces traits of the tensile and impact samples by the scanning electron microscope (SEM) (Made by Carl Zeiss AG, at Jena, Germany).

## 3. Results

### 3.1. Analyses of Process Parameters

#### 3.1.1. The Back Shielding Gas Flow Rate

**Figure 6:**
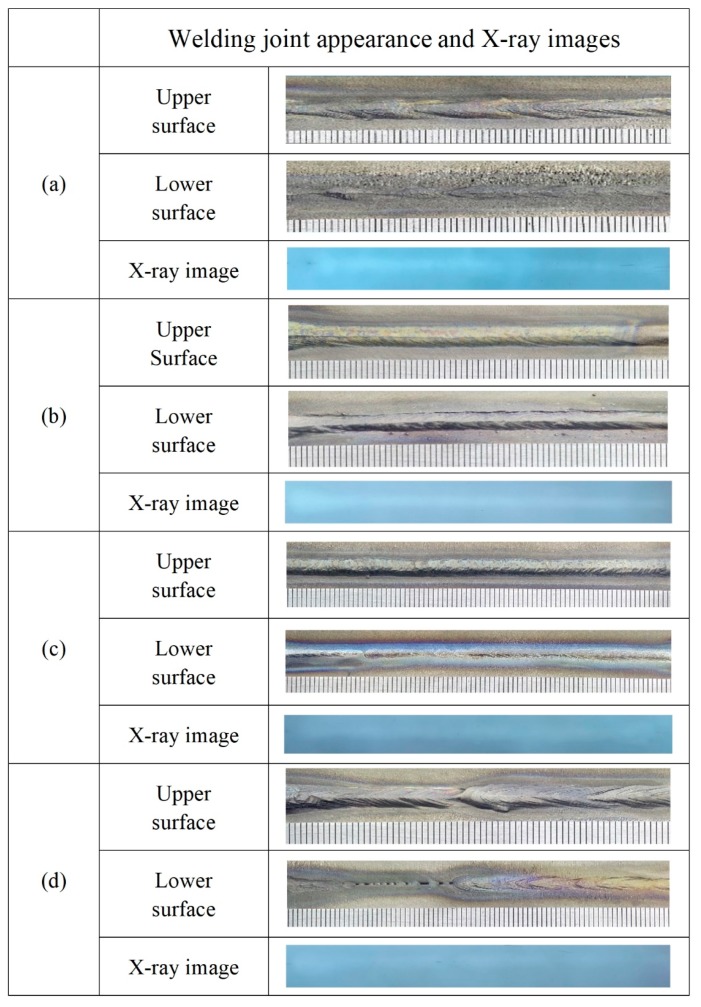
The welding surface appearance and X-ray images at the different BSGFR; (**a**) 15 L/min; (**b**) 25 L/min; (**c**) 35 L/min; (**d**) 45 L/min.

Figure 6 shows the welding surface appearance and X-ray images at the different BSGFR, while the focal position and laser power were kept at 0 mm and 3 kW consistently. With increasing the BSGFR, the protective effect on the weld metal became strong gradually. Then the BSGFR were 15 L/min and 25 L/min, Ti-6Al-4V alloy reacted strongly with oxygen, resulting in serious oxidation on the back of the weld metal. With the BSGFR increased to 35 L/min, the welding surface appearance was more uniform and almost without oxidation. However, as the BSGFR increased further, although the weld metal could be well protected from being oxidized, the welding surface appearance was inhomogeneous on account of the excessive BSGFR that made the water enter into the weld zone. In addition, these weld metals were investigated by X-ray inspection, respectively, the results showed that there were all without welding cracks and pores in the interior of the weld metals under the BSGFR of 15 L/min, 25 L/min, and 35 L/min, but with welding pores in the weld metal at the BSGFR of 45 L/min.

**Figure 7:**
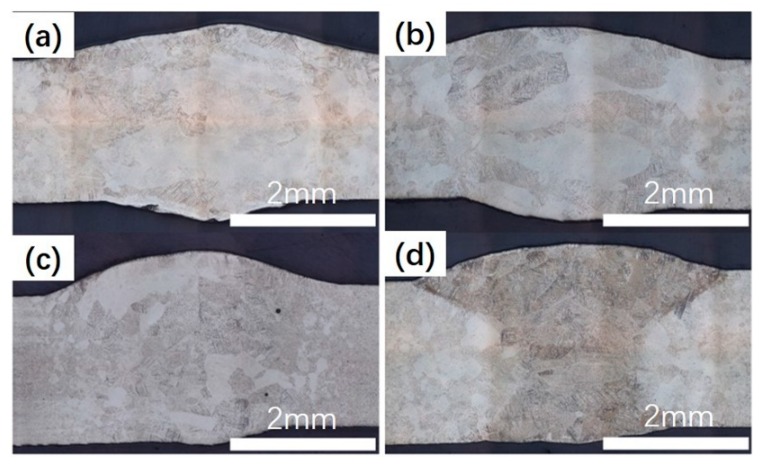
The cross section of welded joints at the different BSGFR; (**a**) 15 L/min; (**b**) 25 L/min; (**c**) 35 L/min; (**d**) 45 L/min.

The influence of the BSGFR on the cross section of welded joints was given in Figure 7. The grain size was calculated via optical microscope software, when the BSGFR was 15 L/min, the average diameter of equiaxial crystals was 342 μm, the average width and length of columnar crystals were 208 μm and 759 μm, respectively. With the BSGFR increased to 25 L/min, the average diameter of equiaxial crystals decreased to 272 μm, meanwhile, the average width and length of columnar crystals reduced to 182 μm and 663 μm. Then the BSGFR further increased to 35 L/min and 45 L/min, the average diameter of equiaxial crystals turned into 114 μm and 92 μm, the average width and length of columnar crystals became 168 μm, 132 μm and 548 μm, 522 μm. Therefore, with the increase of the BSGFR, the grain size became small.

**Figure 8:**
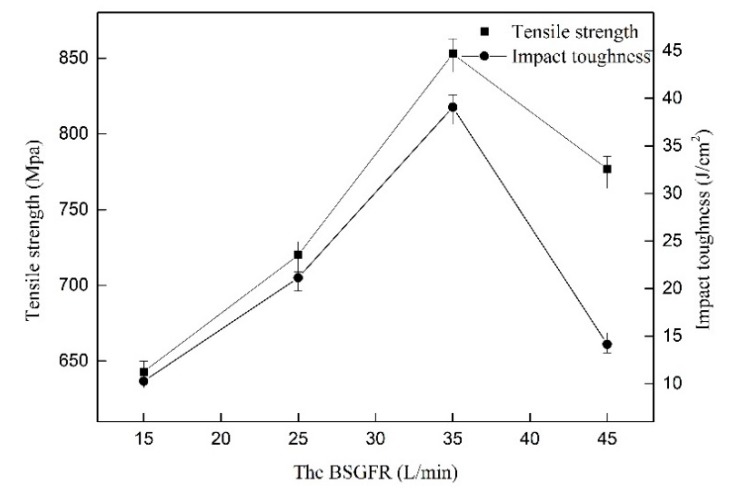
The effect of the BSGFR on tensile strength as well as impact toughness.

Figure 8 shows the tensile strength of the welded joints as well as the impact toughness at the different BSGFR. The tensile strength and impact toughness of the welded joints increased accompanied by the BSGFR increased in the scope of 15 L/min to 35 L/min. However, when the BSGFR exceeded 35 L/min, the mechanical properties began to decline. This was because the increase of the BSGFR sped up the cooling rate of the weld metal, resulting in grain refinement in the weld metal. However, on account of the residual water on the back of the weld metal, water vapor entered the molten pool from the back of the weld. Zhan et al. pointed out that the insoluble gases were difficult to get out adequately of the molten pool, resulting in forming the porosity [21]. The water vapor, as a kind of insoluble gas, when the cooling rate was too large, could not separate out, thus forming pores in the weld metals. Pessoa et al. demonstrated that the lower amount of porosity could improve the mechanical properties [11]. When the BSGFR was 35 L/min, the tensile strength value was maximum, as well as the impact toughness. The tensile strength (852.81 MPa) was up to 95.3% of the base metal, the impact toughness (39.07 J/cm^2^) reached to 78.4% of the base metal. 

#### 3.1.2. Focal Position

**Figure 9:**
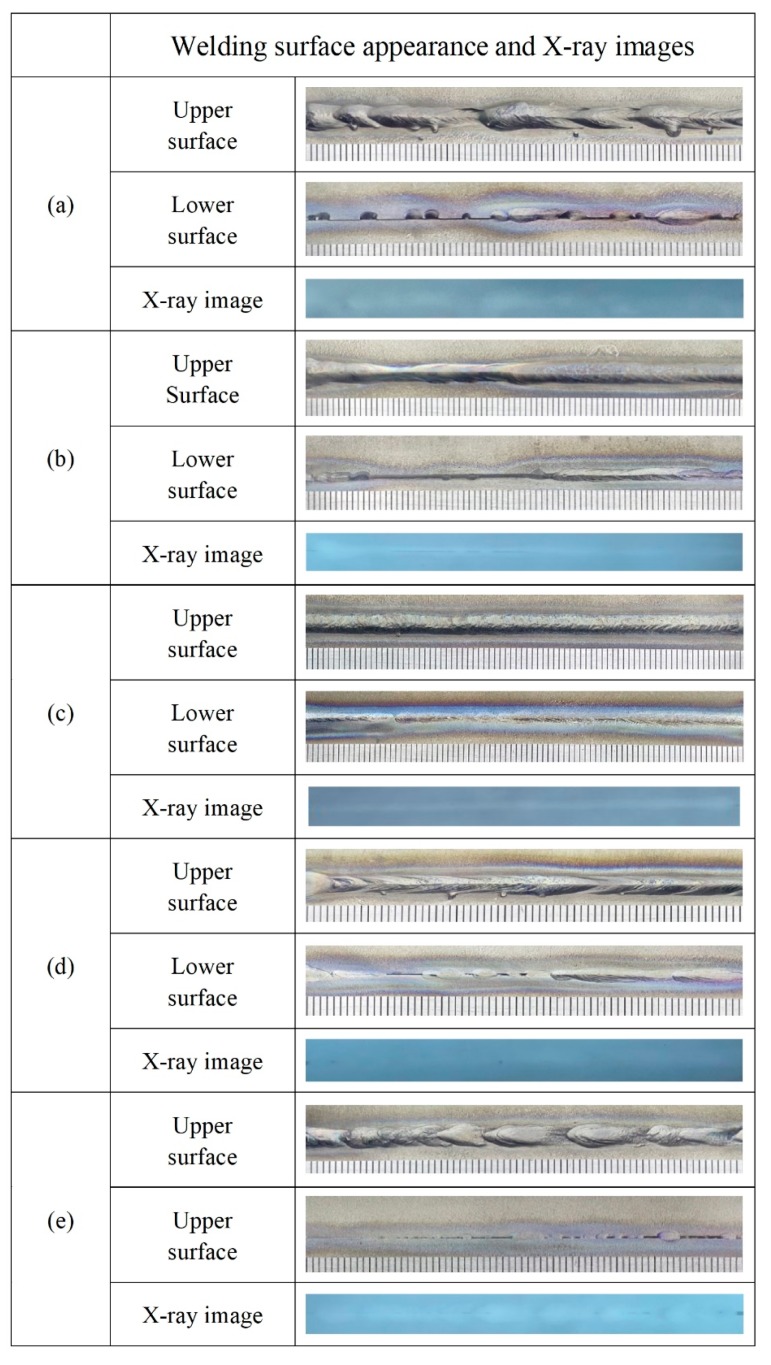
The welding surface appearance and X-ray images at different focal positions; (**a**) −2 mm; (**b**) −1 mm; (**c**) 0 mm; (**d**) +1 mm; (**e**) +2 mm.

The effect of focal position on the welding surface appearance and internal quality were manifested in Figure 9. As the focal position changed from −2 mm to 2 mm, the welding surface appearance became better, firstly, and then worse. When the focal positions were −2 mm and 2 mm, the continuity of the weld metals were poor, and there were not fully penetrated welds. When the focal position gradually changed to 0 mm, the weld metal was not only completely penetrated, but also continuous and stable. At a lower focal position, the degree of energy concentration was high, and a large amount of metal steam would be generated in the molten pool, which would impact the back wall of the molten pool and make the molten pool oscillate, causing some small splashes and poor forming performance. As the focal position was large, the heat input was low, and the weld metal was not enough to melt completely. Therefore, it was easier to form a higher quality of welded joint at a moderate focal position. Besides, there were all without welding cracks and pores in the interior of the weld metals by X-ray inspection.

**Figure 10:**
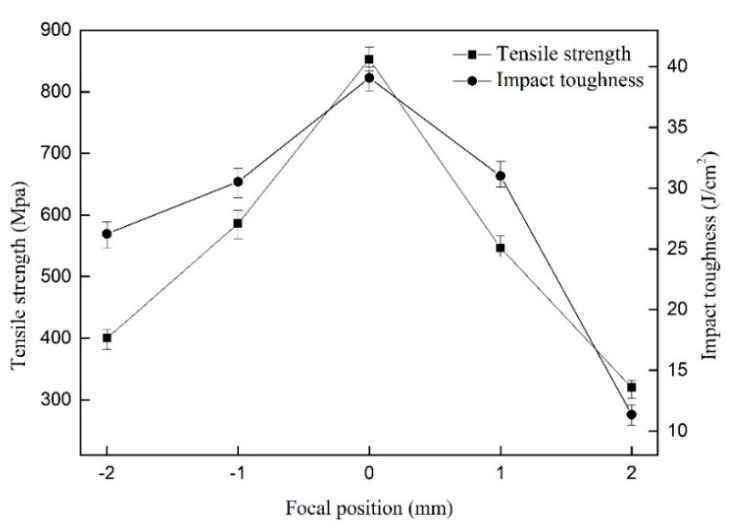
The influence of focal positions on tensile strength as well as impact toughness.

Figure 10 shows the influence of focal positions on the tensile strength of the welded joints as well as the impact toughness. In the process of increasing focal position, the tensile strength as well as impact toughness of the welded joint augmented firstly and then reduced. This was because when the focal position was small, the weld consisted of over-penetration microstructure. Too large focal position led to forming not fully penetrated weld metals. When the focal position was 0 mm, the tensile strength and impact toughness reached the maximum value, the tensile strength value was 852.81 MPa, and the impact toughness was 39.07 J/cm^2^.

#### 3.1.3. Laser Power

**Figure 11:**
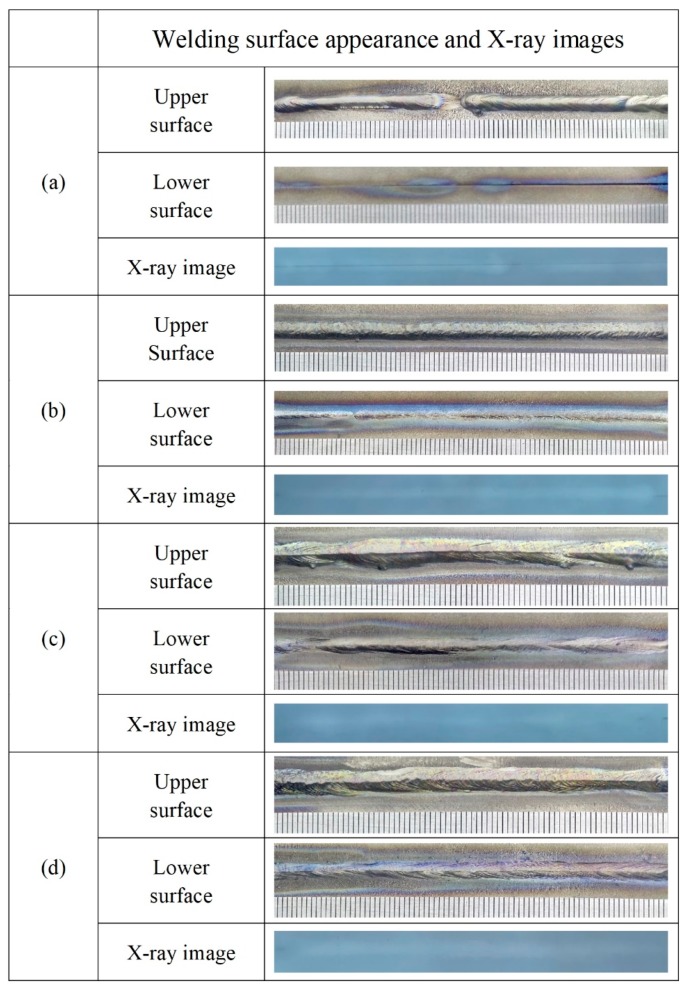
The welding surface appearance and X-ray images at different laser powers; (**a**) 2.5 kW; (**b**) 3 kW; (**c**) 3.5 kW; (**d**) 4 kW.

The welding surface appearance and internal quality were researched under different laser powers, as shown in Figure 11. With the laser power augmented continuously, the width of the weld metal increased gradually, as well as the degree of oxidation, on account of the increased heat input. When the laser power was 2.5 kW, the continuity of the weld metal was poor, and there were not fully penetrated weld metals. This was because the laser energy absorbed on the back of the weld metal was too low to melt. As the laser power reached to 3.0 kW gradually, the weld metal was completely fused. As the increase of laser power further, the back width of the weld metals also kept increasing and the weld metals were oxidized severely. There were all without welding cracks and pores in the interior of the weld metals via X-ray inspection.

**Figure 12:**
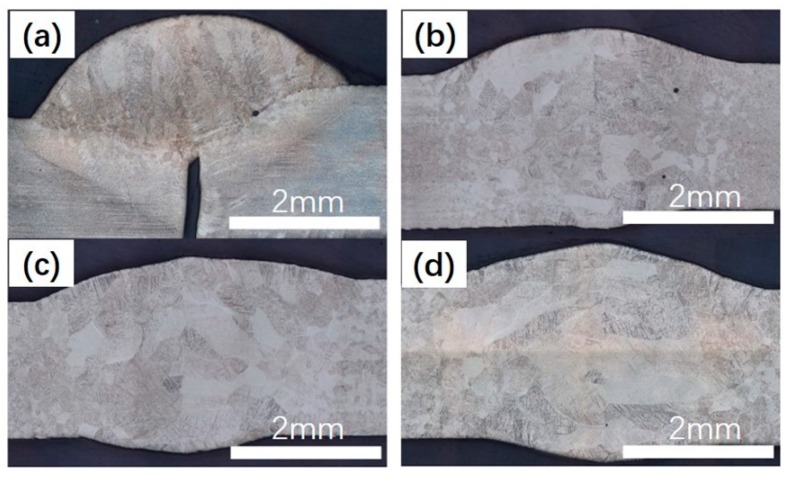
The cross section of welded joints at different laser powers; (**a**) 2.5 kW; (**b**) 3 kW; (**c**) 3.5 kW; (**d**) 4 kW.

The influence of laser power on the crosssection of welded joints was manifested in Figure 12. When the laser power was 2.5 kW, the weld metals were not fully penetrated. As the laser power were 3.0 kW, 3.5 kW, and 4.0 kW, respectively, the average diameter of equiaxial crystals were 114 μm, 174 μm, and 201 μm The average width of columnar crystals were 168 μm, 195 μm, and 259 μm, and the average length were 548 μm, 743 μm, and 764 μm. Therefore, with the increase of laser power, the grain size became larger.

**Figure 13:**
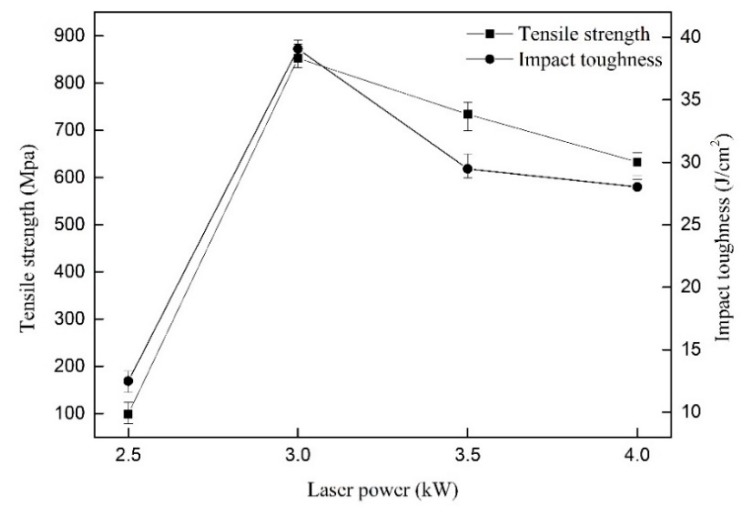
The influence of laser powers on tensile strength and impact toughness.

Figure 13 shows the influence of laser powers on the tensile strength of welded joints as well as impact toughness. As the laser power kept increasing, the tensile strength of welded joints augmented firstly and then decreased, as well as the impact toughness. When the laser power was low, the weld metals were not fully penetrated. However, the laser power was too large, resulting in excessive grain size, which made the tensile strength reduce, as reported by Wang [22]. As the laser power was 3.0 kW, the tensile strength and impact toughness achieved the maximum value simultaneously, they were 852.81 MPa and 39.07 J/cm^2^.

### 3.2. Investigation on the Welding Quality

Underwater and In-air in-air LBW with filler wire experiment of Ti-6Al-4V alloy at the optimum process parameters were observed. By comparing the welding surface appearance, microstructure, as well as the mechanical properties, ULBW with filler wire of Ti-6Al-4V alloy was better evaluated, the optimum process parameters were listed in Table 4. 

**Figure 14:**
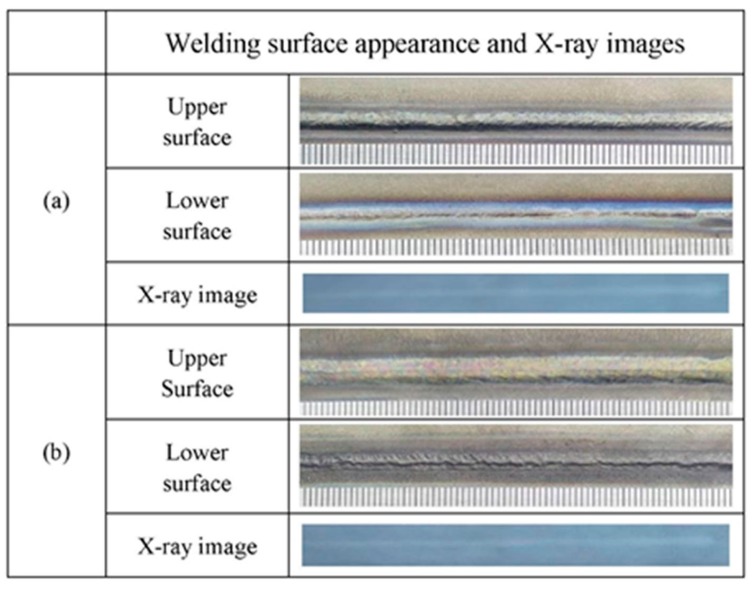
The welding surface appearance and X-ray images; (**a**) the underwater weld metal; (**b**) the in-air weld metal.

Figure 14 shows the welding surface appearance and X-ray images of Ti-6Al-4V alloy via LBW with filler wire. The welding surface appearance of the underwater and in-air weld metals were both continuous and uniform. In addition, the underwater and in-air weld metals were investigated by X-ray inspection, respectively. There were without welding cracks and pores in the interior of the underwater and in-air weld metals.

**Figure 15:**
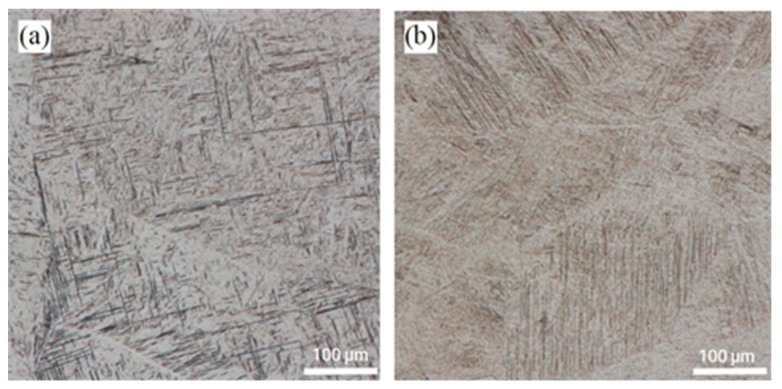
The microstructure of the fusion zones; (**a**) the underwater weld metal; (**b**) the in-air weld metal.

The microstructures in the fusion zones of the underwater and in-air weld metals were shown in Figure 15. Firstly generated columnar crystals in the fusion line near the base metal and then gradually grew to the center of the weld metals. The microstructure in the fusion zones of the underwater and in-air weld metals was acicular martensite. They distributed mainly in the form of intersecting perpendicular “basket” in the underwater weld metals, whereas were scattered disorderly in the in-air weld metals.

**Figure 16:**
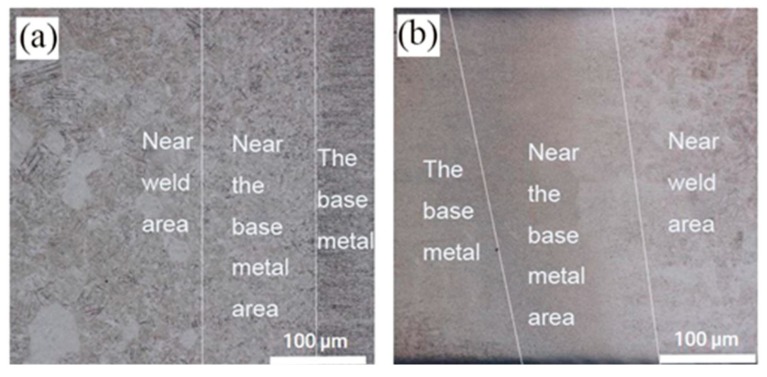
The microstructures of the heat affected zones; (**a**) the underwater weld metal; (**b**) the in-air weld metal.

The microstructure in heat affected zones of the underwater and in-air weld metals was exhibited in Figure 16. The heat affected zone could be mainly divided into two diverse areas in the underwater and in-air weld metals, they were near the fusion zone and base metal zone, the former consisted of the intersecting perpendicular “basket” structure of α + α′ phase, the latter was mainly composed of the coarse α and β phase, almost without α′ phase. Because the temperature of the former was higher than the latter, more β phases transformation occurred.

**Figure 17:**
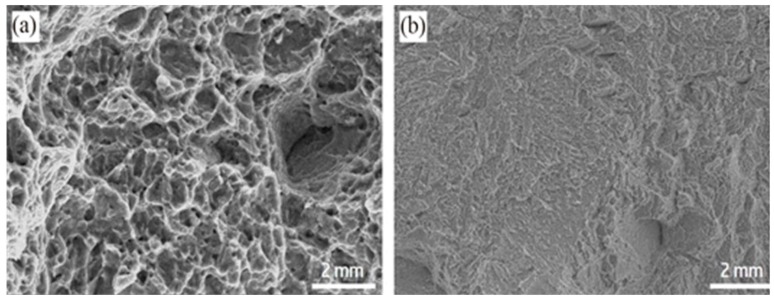
Images of the tensile fracture surfaces; (**a**) the underwater welded joint; (**b**) the in-air welded joint.

**Figure 18:**
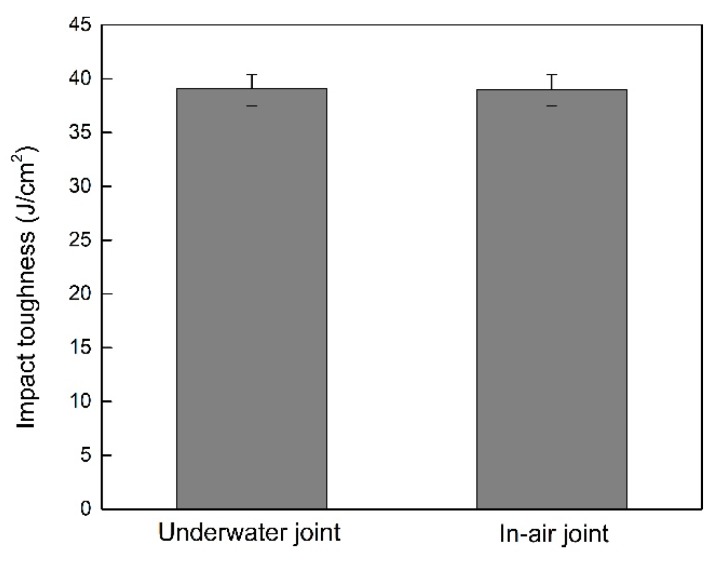
The impact toughness of the underwater and in-air welded joints.

**Figure 19:**
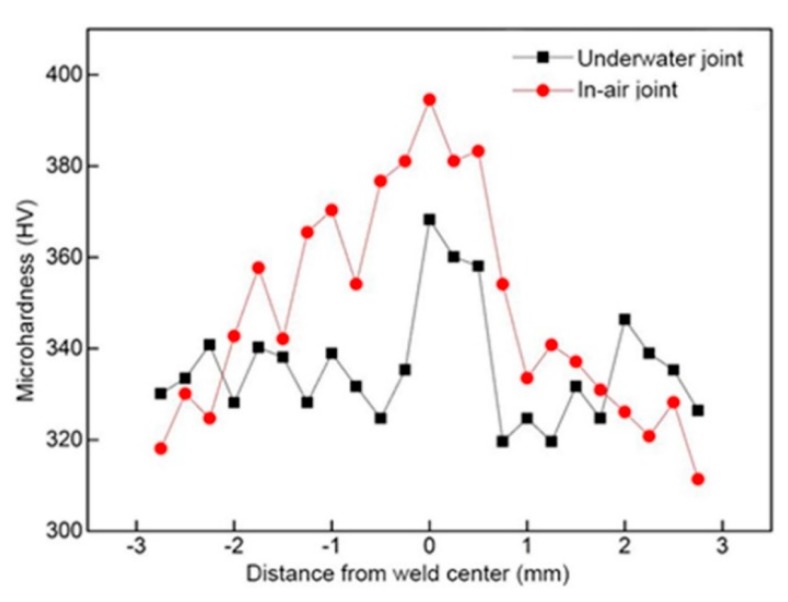
The microhardness of the underwater and in-air welded joints.

Through the tensile tests of samples, both of them were fractured at the weld metals. The tensile strength of the in-air welded joints was 861.32 MPa, and that of the underwater welded joints was 852.81 MPa, which could fill the bill of the applications. The fracture surfaces of tensile specimens were researched by SEM. As shown in Figure 17. It can be found that both of them were mixed fractures of quasi-cleavage fracture and dimple fracture. As displayed in Figure 18, the impact toughness of the underwater welded joints was 39.07 J/cm^2^ and that of the in-air welded joints was 38.99 J/cm^2^, both of them were lower than that of the base metal (49.85 J/cm^2^). It was mainly because of Ti-6Al-4V alloy with good plasticity, after welding, the as-cast organization formed in the weld metals. The microhardness of the welded joints was investigated. As exhibited in Figure 19, for the in-air and underwater welded joints, the microhardness value of fusion zones and heataffected zones compared with that of base metal had large ascension, besides, the microhardness value was the highest in the fusion zones, and it gradually degraded from the fusion zones to the heataffected zones. Because the microstructure of the fusion zones was mainly acicular martensite α′ phase that made the microhardness increase, the α′ phase gradually decreased from the fusion zones to the heataffected zones, which led to decrease of the microhardness value. In addition, the base metal microhardness value was the lowest owing to without α′ phase. The microhardness values were basically the same for the underwater and in-air welded joints, so the welded joints did not harden obviously by using the ULBW with filler wire.

## 4. Conclusions

(1) When the BSGFR was 35 L/min, the focal position was 0 mm, and the laser power was 3.0 kW, the welding surface appearance was continuous and mechanical properties were also optimized.

(2) In the fusion zones, acicular martensite distributed mainly in the form of intersecting perpendicular “basket” in the underwater weld metals, but they were scattered disorderly in the in-air weld metals.

(3) For the underwater as well as the in-air welded joints, the near the fusion zones consisted of the α + α′ phases, whereas the near base metal zones were mainly composed of the coarse α and β phases.

(4) The tensile strength of the underwater welded joints (852.81 MPa) reached to 95.26% of the base metal (895.24 MPa). The impact toughness of the underwater welded joints was 39.07 J/cm^2^, and 78.38% of the base metal (49.85 J/cm^2^).

## Figures and Tables

**Table 1:** TC4 titanium alloy chemical component (mass fraction/%).

C	O	H	N	Al	V	Fe	Ti
0.06	0.12	0.009	0.03	6.4	4.2	0.18	Bal.

**Table 2:** TC4 titanium alloy mechanical properties.

Tensile Strength (MPa)	Yield Strength (MPa)	Elongation (%)	Impact Toughness (J/cm^2^)
895.24	835.49	8.17	49.85

**Table 3:** Process parameters.

Welding Parameters	BSGFR (L/min)	Focal Position (mm)	Laser Power (kW)	Welding Speed (mm/s)	Wire Feed Speed (mm/s)
Value	15–45	−2–+2	2.5–4	20	60

**Table 4:** Welding test parameters.

Test	Water Depth (mm)	Laser Power (kW)	Welding Speed (mm/s)	Wire Feeding Speed (mm/s)	Focal Position (mm)	BSGFR (L/min)
Underwater	60	3.0	20	60	0	35
In-air	0	3.0	20	60	0	35
